# Association Between Perceived Levels of Stress and Self-Reported Food Preferences Among Males and Females: A Stated Preference Approach Based on the China Health and Nutrition Survey

**DOI:** 10.3389/fpubh.2022.850411

**Published:** 2022-03-25

**Authors:** Fahui Yang, Ruonan Li, Xiaojian Ren, Bing Cao, Xiao Gao

**Affiliations:** ^1^Key Laboratory of Cognition and Personality, Faculty of Psychology, Ministry of Education, Southwest University, Chongqing, China; ^2^National Demonstration Center for Experimental Psychology Education, Southwest University, Chongqing, China; ^3^Zibo Central Hospital, Zibo, China

**Keywords:** perceived stress, food preference, eating behavior, population-based study, dietary

## Abstract

**Objective:**

Stress is a major public health challenge and is associated with undesirable eating behavior. This cross-sectional study aimed to explore whether there is an association between perceived level of stress and food preference among Chinese adults.

**Study Design:**

Perceived level of stress was measured using the Chinese version of the 14-item Perceived Stress Scale, whereas self-reported food preferences were investigated by means of five food classification questions, including questions regarding the consumption of fast food, salty snacks, fruit, vegetables, and soft/sugary drinks.

**Methods:**

The data were collected from the 2015 China Health and Nutrition Survey. Information of 8,216 adults (≥18 years) on perceived level of stress, self-reported food preferences, and other important covariates was available and analyzed.

**Results:**

Perceived level of stress was negatively associated with a preference for fruit (β = −0.58, 95% CI: −0.81 to −0.34, *p* < 0.0001) and vegetables (β = −1.13, 95% CI: −1.41 to −0.85, *p* < 0.0001), while it was positively associated with a preference for fast food (β = 0.36, 95% CI: 0.08–0.64, *p* = 0.011) and soft/sugary drinks (β = 0.48, 95% CI: 0.30–0.66, *p* < 0.0001) after adjusting for potential confounders. No association between a preference for salty snacks and perceived level of stress was found in either men or women.

**Conclusions:**

The present population-based study reported strong associations between perceived level of stress and self-reported food preferences among Chinese adults. Sex differences related to this association were also worthy of attention.

## Introduction

Stress is considered a major public health challenge in modern society; it increases the risk of obesity, metabolic syndrome, and various other chronic diseases (1, 2). Simultaneously, stress-related eating is a potential factor in the obesity pandemic (3, 4). Food preferences are influenced by a myriad of environmental and internal factors, which include the availability and rewarding properties of food (5, 6). Consuming highly tasty foods stimulates the food reward system, and the pleasurable experience increases the likelihood of repeat consumption, potentially leading to overweight and obesity (7).

Previous studies have indicated that exposure to stress disrupts eating patterns; individuals may attempt to relieve stress by engaging in unhealthy—although often pleasurable—behaviors, including undesirable eating behavior (8). The association between the emotions related to stress and undesirable eating behavior, such as emotional eating, restrained eating, and external eating, has been reported in several studies (9–11). Both men and women perceive stress as leading to unhealthy changes in eating patterns (12). Evidence from animal studies have shown an increased intake of palatable foods in rats with chronic stress, which may assist in ameliorating anxiety—the alteration of the function of the hypothalamic–pituitary–adrenal axis may be the mediating factor in this association (13). A randomized controlled trial examined the effects of stress on food preferences and obesogenic behavior; however, the findings from this study do not support the hypothesis that perceived stress increases the preference for highly palatable foods (14). Although there is accumulating evidence of a possible association between perceived stress and food preferences and selection, some studies have found that stress suppresses appetite, whereas other studies report that persistent stress promotes the motivation to eat, especially foods with a high energy, sugar, and fat content (15, 16). Therefore, the primary aim of the present study was to determine whether there is an association between perceived level of stress and food preference.

Furthermore, literature indicates that the influence of perceived stress on food choice varies based on sex (17, 18). An epidemiological study found that women with high levels of stress have an increased preference for sweet and high-fat foods compared to their counterparts with low levels of stress (19). However, another study found that men with no perceived stress consume more unhealthy foods than their counterparts with low levels of stress (20). The preference to adopt more indulgent eating behavior in response to stress is especially evident in women, while stress is not associated with indulgent eating behavior in men, regardless of the level of stress (21). Women, more often than men, employ indulgent eating as a stress management strategy, while men tend to follow a problem-focused approach to cope with stress (22). Accordingly, this study proposed to further explore sex differences in stress-induced dietary preferences.

To date, most research on the relationship between stress and food preferences has been conducted in Western countries; few studies focus on this association among China's large population. Chinese eating patterns and food preferences vastly differ from those of Western populations. Additionally, findings from previous studies are inconsistent. Herein, by using the database of the China Health and Nutrition Survey (CHNS), the main objective was to explore the underlying relationship between perceived level of stress and food preferences among males and females in Chinese adults, and to verify whether this association also exists after adjusting for potential confounders.

## Methods

### Data Source and Study Participants

**Figure 1:**
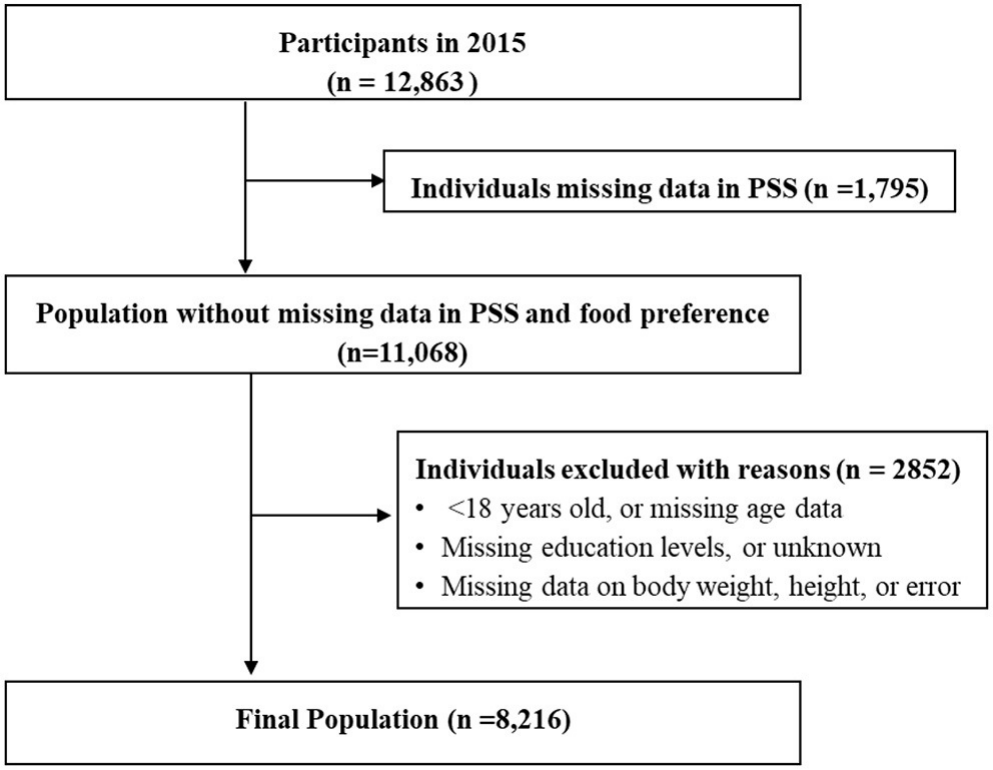
Flow chart of the selection process of study participants from the China Health and Nutrition Survey.

The CHNS comprises a series of population-based longitudinal household surveys conducted by the National Institute for Nutrition and Health of the Chinese Center for Disease Control and Prevention and the University of North Carolina at Chapel Hill in the United States (23). The household surveys cover nine provinces in China that vary substantially in geography, economic development, public resources, and health indicators, which was given to participants by paper questionnaires. The de-identified survey data are publicly available. The survey commenced in 1989 following a multistage, random cluster sampling method. To date, individual-level information on health, socioeconomic status, and social and family networks has been collected in 11 waves (i.e., in 1989, 1991, 1993, 1997, 2000, 2004, 2006, 2009, 2011, 2015, and 2018). The questionnaire was originally in Chinese, which included questions that related to individual activities, lifestyle, health status, marriage and birth history, body shape, and mass media exposure. Our study only selected the variables that we are interested in. The first survey to include the Perceived Stress Scale (PSS) was that conducted in 2015. The results of the 2018 wave were not yet available at the time of the present study; therefore, we used the data of the 2015 wave. In total, 12,863 individuals completed the 2015 CHNS interview. We excluded individuals with an age <18 years, missing PSS data (*n* = 1,795), missing age, height, weight, or education level data, and those with incorrect data concerning any of the abovementioned variables (*n* = 2,852). In total, 8,216 participants were included in the present analysis. Figure 1 presents a detailed flowchart of participant selection. Further information regarding the CHNS project is available at the following URL: https://www.cpc.unc.edu/projects/china.

### Data Collection

Data on all demographic variables, including age, sex, education level, weight (kg), height (m), daily sleep duration (hours), urban status, smoking status, and alcohol consumption status, were extracted from the original CHNS files. Weight and height measurements were obtained by means of detailed physical examinations, and the remaining variables by means of objective questionnaires.

The Chinese version of the 14-item PSS (PSS-14) was used to measure perceived level of stress, with the total score ranging from 0 to 56 (23). The original PSS consists of 14 items; it was translated from English to Chinese and subsequently back to English to ensure the accuracy of translation. Among the 8,216 included participants, we calculated the Cronbach's alpha of the PSS-14 as 0.829, which is similar to the scale reliability findings of Huang et al. calculated among 9,507 individuals from the same database (i.e., 0.830) (23). The scale is divided into two subscales: items 1, 2, 3, 8, 11, 12, and 14 constitute the negative subscale, focusing on the feeling of tension, whereas items 4, 5, 6, 7, 9, 10, and 13 constitute the positive subscale, focusing on the sense of feeling out of control. The negative subscale included questions regarding the frequency of negative incidents or feelings, such as ‘In the last month, how often have you felt that you were unable to control important things in your life?' The positive subscale included positively stated items, e.g., “In the last month, how often have you felt confident about your ability to handle your personal problems?” A 5-point Likert scale was used to assess each item, ranging from 0 = “never” to 4 = “very often”. To obtain meaningful odds ratios, perceived level of stress was rescaled based on the median total PSS-14 score (i.e., a low level and high level of perceived stress).

Self-reported food preferences were investigated by means of five food classification questions, including questions regarding the consumption of fast food (e.g., pizza and hamburgers), salty snacks (e.g., pretzels and potato chips), fruit, vegetables, and soft/sugary drinks (including fruit drinks and soft drinks) (24). For each question, the answer options were: “dislike very much”, “dislike somewhat”, “neutral”, “like somewhat”, or “like very much”.

### Statistical Analyses

According to the Chinese Working Group on Obesity (25), body mass index (BMI) was divided into the following groups: underweight, normal, overweight, and obese representing a BMI <18.5, 18.5–23.99, 24–27.99, and ≥28 kg/m^2^, respectively. Data are presented as mean (standard deviation [SD]) or median (interquartile range) for continuous variables, and as percentages for categorical variables. For continuous variables, independent sample *t*-tests or one-way analysis of variance were used for comparisons between two or more independent groups. For categorical variables, the χ^2^-test was used to assess the statistical significance of differences between various groups. The Bonferroni correction was used to reduce the chances of obtaining false-positive results (type I errors) when multiple pairwise tests were performed on a single set of data. Linear regression models were used to investigate the association between food preferences and PSS scores. Regression coefficients (β) and their 95% confidence intervals (CIs) were estimated. The association between the risk of high levels of perceived stress and food preferences was assessed using logistic regression models. The maximum likelihood method was used to estimate odds ratios and their 95% CIs (26). We adjusted for the confounders of basic characteristics (i.e., age, sex, education, and urban status) and physical conditions (i.e., BMI, smoking status, alcohol consumption status, and the presence of diabetes or hypertension) in the linear and logistic regression models; these were selected based on previous studies following a similar study design (27, 28). Most potential confounders are dichotomous or continuous variables. Only education is multicategorical variable, the first level (i.e., Grad from primary) is considered as reference. We used the variance inflation factor to detect potential multicollinearity in the regression models. A two-tailed *p* < 0.05 was considered statistically significant. All statistical tests were performed using Stata Statistical Software Release 15 (Stata Corp LLC, College Station, TX, USA).

## Results

### Basic Participant Characteristics

Table 1 presents the basic characteristics of the sample population, stratified by sex. The 2015 CHNS wave included 8,216 adults (≥18 years) with information on perceived level of stress, self-reported food preferences, and the remaining relevant covariates (Figure 1). In total, 4,197 (51.1%) participants were women and the mean age was 50.8 years (SD = 14.2). The education level of more than two-thirds of the participants was lower than high-school level education, and most (64.7%) were from urban areas. Approximately half of the participants were overweight (36.1%) or obese (14.4%), while the prevalence of diabetes and hypertension was 4.9 and 16.0%, respectively. Apart from the prevalence of diabetes, all variables differed significantly between men and women (*p* < 0.05).

**Table 1:** The basic characteristics of selected participants.

**Variables**	**Total** **(*N* = 8,216)**	**Male ** **(*n =* 4,019)**	**Female** **(*n =* 4,197)**	*p*-value^*^
**Age (years, mean** **±SD; n, %)**	50.8 ± 14.2	52.2 ± 14.4	49.5 ± 14.0	<0.0001 ^a^
**Education (n, %)**				
Grad from primary	1,601 (19.5)	705 (17.5)	896 (21.4)	<0.0001 ^b^
Lower middle school degree	3,124 (38.0)	1,538 (38.3)	1,586 (37.8)	
Upper middle school degree	1,386 (16.9)	726 (18.1)	660 (15.7)	
Technical or vocational degree	794 (9.7)	378 (9.4)	416 (9.9)	
University or college degree and above	1,311 (16.0)	672(16.7)	639 (15.2)	
**Urban Status (** ***n*** **, %)**				
City, town, or county capital city	4,490 (64.7)	2,292 (57.0)	2,198 (52.4)	<0.0001 ^b^
Suburban or rural village	3,726 (45.4)	1,727 (43.0)	1,999 (47.6)	
**BMI (kg/m**^**2**^**, mean** **±SD;** ***n*****, %)**	24.3 ± 3.6	24.5 ± 3.6	24.1 ± 3.7	<0.0001 ^a^
Underweight	362 (4.4)	150 (3.7)	212(5.1)	<0.0001 ^b^
Normal	3,702 (45.1)	1,726 (42.9)	1,976 (47.1)	
Overweight	2,967 (36.1)	1,542 (38.4)	1,425 (33.9)	
Obese	1,185 (14.4)	601 (14.9)	584 (13.9)	
**Smoking status (** ***n*** **, %)**				
Yes	1,850 (22.8)	1,774 (45.1)	76 (1.8)	<0.0001 ^b^
No	6,280 (77.2)	2,163 (54.9)	4,117 (98.2)	
**Alcohol consumption (** ***n*** **, %)**				
Yes	2,257 (28.7)	2,301 (52.6)	226 (5.7)	<0.0001 ^b^
No	5,600 (71.3)	1,827 (47.4)	3,773 (94.3)	
**Diabetes (** ***n*** **, %)**				
Yes	382 (4.9)	202 (5.3)	180 (4.5)	0.127 ^b^
No	7,461 (95.1)	3,647 (94.7)	3,814 (95.5)	
**Hypertension (** ***n*** **, %)**				
Yes	1,254 (16.0)	664 (17.2)	590 (14.8)	0.003 ^b^
No	6,593 (84.0)	3,187 (82.8)	3,406 (85.2)	

* *p-value refers to the comparison between male and female*.

a *P-values were calculated by two-tailed t-tests*.

b *P-values were calculated by chi-square tests*.

### Association Between Perceived Level of Stress and Basic Participant Characteristics

The associations between perceived level of stress and basic participant characteristics are presented in Table 2. Participants who differed in age group, education level, and weight category had different levels of perceived stress (*p* < 0.05). Additionally, those who resided in cities, towns, or county capital cities experienced higher levels of perceived stress than those who resided in suburban or rural villages (PSS-14 score: 22.9 vs. 22.3). Unexpectedly, participants who consumed alcohol and had hypertension experienced reported lower levels of perceived stress (both *p* < 0.05). We did not find differences in perceived stress levels between participants with or without diabetes (*p* = 0.071). The comparison of low and high levels of perceived stress based on the basic characteristics of the participants is presented in Supplementary Table 1. The details of original results are shown in Supplemental Table 2.

**Table 2:** The comparison of perceived stress with different basic characteristics of participants.

**Variables**	***n* (%)**	**PSS-14 scores**	***p*-value**
**Age (years)**			
18–40	1,996 (24.3)	22.9 ± 5.9	0.011
41–59	3,790 (46.1)	22.7 ± 6.2	
≥60	2,430 (29.6)	22.4 ± 6.3	
**Sex (** ***n*** **, %)**			
Male	4,019 (48.9)	22.6 ± 6.1	0.279
Female	4,197 (51.1)	22.7 ± 6.2	
**Education (** ***n*** **, %)**			
Grad from primary	1,601 (19.5)	23.2 ± 6.1	<0.0001
Lower middle school degree	3,124 (38.0)	23.0 ± 6.0	
Upper middle school degree	1,386 (16.9)	22.4 ± 6.0	
Technical or vocational degree	794 (9.7)	22.0 ± 6.1	
University or college degree and above	1,311 (16.0)	21.8 ± 6.4	
**Urban**			
1-city, town or county capital city	4,490 (54.6)	22.9 ± 6.1	<0.0001
0-suburban or rural village	3,726 (45.4)	22.3 ± 6.1	
**Weight category**			
Underweight	362(4.4)	23.4 ± 6.1	0.007
Normal	3,702 (45.1)	22.7 ± 6.0	
Overweight	2,967(36.1)	22.6 ± 6.3	
Obese	1,185 (14.4)	22.2 ± 6.2	
**Smoking status**			
Yes	1,850 (22.8)	22.5 ± 6.1	0.318
No	6,280 (77.2)	22.6 ± 6.2	
**Alcohol consumption**			
Yes	2,257 (28.7)	22.3 ± 6.1	0.011
No	5,600 (71.3)	22.7 ± 6.2	
**Diabetes**			
Yes	382 (4.9)	22.0 ± 6.7	0.071
No	7,461 (95.1)	22.6 ± 6.2	
**Hypertension**			
Yes	1,254 (16.0)	22.1 ± 6.5	0.002
No	6,593 (84.0)	22.7 ± 6.1	

### Association Between Perceived Level of Stress and Food Preferences

No potential multicollinearity was detected in the regression models. From the non-adjusted models, it was found that the PSS-14 score was negatively associated with a preference for fruit (β = −0.61, 95% CI: −0.83 to −0.38, *p* < 0.0001) and vegetables (β = −1.18, 95% CI: −1.45 to −0.91, *p* < 0.0001), while it was positively associated with a preference for soft/sugary drinks (β = 0.52, 95% CI: 0.35–0.69, *p* < 0.0001). The results remained similar for the abovementioned food preferences (fruit, vegetables, and soft/sugary drinks) after adjusting for confounders of basic characteristics (i.e., age, sex, education, and urban status) in Model 1, and those of basic characteristics and physical conditions (i.e., BMI, smoking status, alcohol consumption status, and presence of diabetes or hypertension) in Model 2. A preference for fast food was positively associated with the PSS-14 score in Model 1 (β = 0.31, 95% CI: 0.04–0.58, *p* = 0.026) and Model 2 (β = 0.36, 95% CI: 0.08–0.64, *p* = 0.011). The details are presented in Table 3. The logistic regression results, reflecting the association between a high level of perceived stress and food preferences, are presented in Supplementary Table 3. We found that a high level of perceived stress was associated with a preference for fast food, salty snacks, and soft/sugary drinks after adjusting for the abovementioned confounders. Moreover, a low level of perceived stress was associated with a preference for fruit and vegetables in the adjusted model.

**Table 3:** The associations between PSS-14 scores and each variable of food preference.

**Variables**	Model^*^	**β (95%CI)**	***t***	***p*-value**
**Fast food**	**Non-adjusted model**	0.27 (0.00, 0.54)	1.93	0.053
	**Model 1**	0.31 (0.04,0.58)	2.23	0.026
	**Model 2**	0.36 (0.08, 0.64)	2.55	0.011
**Salty snack food**	**Non-adjusted model**	0.08 (−0.18, 0.34)	0.62	0.537
	**Model 1**	0.08 (−0.18, 0.35)	0.63	0.529
	**Model 2**	0.07 (−0.20, 0.33)	0.48	0.631
**Fruits**	**Non-adjusted model**	−0.61 (−0.83, −0.38)	−5.20	<0.0001
	**Model 1**	−0.58 (−0.81, −0.35)	−4.90	<0.0001
	**Model 2**	−0.58 (−0.81, −0.34)	−4.73	<0.0001
**Vegetables**	**Non-adjusted model**	−1.18 (−1.45, −0.91)	−8.59	<0.0001
	**Model 1**	−1.17 (−1.44, −0.90)	−8.53	<0.0001
	**Model 2**	−1.13 (−1.41, −0.85)	−7.95	<0.0001
**Soft/sugared drinks**	**Non-adjusted model**	0.52 (0.35, 0.69)	6.03	<0.0001
	**Model 1**	0.51 (0.34,0.69)	5.84	<0.0001
	**Model 2**	0.48 (0.30, 0.66)	5.30	<0.0001

* *Model 1: adjusted for age, sex, urban status, education*.

*Model 2: model 1 + BMI+ smoking status + alcohol consumption + diabetes + hypertension*.

### Sex Differences in the Association Between Perceived Level of Stress and Food Preferences

The results regarding the association between the PSS-14 score and food preferences in men compared with women are presented in Table 4. No association was found between a preference for salty snacks and perceived levels of stress in either men or women. A preference for fast food was positively associated with perceived level of stress in women after adjusting for potential confounders, including age, sex, education level, urban status, BMI, smoking status, alcohol consumption status, and presence of diabetes or hypertension (β = 0.46, 95% CI: 0.08–0.85, *p* = 0.019). The association between a preference for fruit and perceived level of stress was also significant in women (β = −0.93, 95% CI: −1.30 to −0.57, *p* < 0.0001), although it was not significant in men (*p* > 0.05), after adjusting for the abovementioned confounders. Moreover, perceived level of stress showed a significant negative association with a preference for vegetables, and a significant positive association with a preference for soft/sugary drinks, in both men and women before and after adjusting for potential confounders (all *p* < 0.05).

**Table 4:** Associations between PSS-14 scores and Each Variable of Food Preference in Male and Female.

**Variable**	Model^*^	**Male**			**Female**		
		**β(95%CI)**	***z***	***p*-values**	**β(95%CI)**	***z***	***p*-values**
**Fast food**	**Non-adjusted model**	0.22 (−0.17, 0.62)	1.12	0.264	0.30 (−0.07, 0.68)	1.60	0.111
	**Adjusted model**	0.25 (−0.16, 0.65)	1.20	0.231	0.46 (0.08, 0.85)	2.35	0.019
**Salty snack food**	**Non-adjusted model**	0.34 (−0.04, 0.72)	1.75	0.081	−0.16 (−0.51, 0.20)	−0.85	0.397
	**Adjusted model**	0.37 (−0.03, 0.76)	1.83	0.068	−0.18 (−0.55, 0.19)	−0.97	0.334
**Fruits**	**Non-adjusted model**	−0.38 (−0.68, −0.07)	−2.44	0.015	−0.97 (−1.32, −0.62)	−5.38	<0.0001
	**Adjusted model**	−0.31 (−0.62, 0.01)	−1.92	0.055	−0.93 (−1.30, −0.57)	−4.97	<0.0001
**Vegetables**	**Non-adjusted model**	−1.18 (−1.55, −0.81)	−6.27	<0.0001	−1.14 (−1.53, −0.74)	−5.65	<0.0001
	**Adjusted model**	−1.14 (−1.52, −0.75)	−5.83	<0.0001	−1.08 (−1.49, −0.67)	−5.17	<0.0001
**Soft/sugared drinks**	**Non-adjusted model**	0.39 (0.15, 0.63)	3.17	0.002	0.64 (0.40, 0.88)	5.25	<0.0001
	**Adjusted model**	0.35 (0.10, 0.61)	2.71	0.007	0.58 (0.33, 0.83)	4.58	<0.0001

* *Adjusted model, adjusting for age, sex, education, urban status, BMI, smoking status, alcohol consumption, diabetes, and hypertension*.

## Discussion

Using the large database of the 2015 CHNS, our study provides valuable evidence regarding the association between food preferences and perceived levels of stress among the Chinese population. Moreover, sex differences related to this association were also explored in this study. We found that perceived levels of stress were associated with unhealthy food preferences, including increased intake of fast food and soft/sugary drinks and decreased intake of fruit and vegetables. Perceived levels of stress were positively associated with a preference for fast food and negatively associated with a preference for fruit in women, but not men, in the adjusted models.

Our findings are similar to those from previous Western studies in that a higher perceived level of stress is associated with a preference for highly tasty foods (14). Various studies have reported that perceived levels of stress influence specific food preferences and alter food choices (29, 30). For example, some researchers suggest that stress induces people to eat in the absence of hunger and to choose higher energy foods, which may be related to the food reward system (29–31). Consistent with our findings, several original studies exploring the foods that individuals tend to consume under stress have confirmed that highly caloric fatty snacks, sweets, and foods are preferred (32–34). Accumulating evidence suggests that increased stress levels are associated with decreased consumption of fruit and vegetables (35–37). Similarly, we found that perceived levels of stress were negatively associated with a preference for a healthy dietary taste (i.e., fruit and vegetables). Overall, both our current findings and those of multiple previous studies demonstrated that chronic stress is positively associated with the frequency of emotional eating, snacking, and consuming tasty foods, and negatively associated with consuming fruit and vegetables (35–37). However, not all opinions support our findings. For example, a cross-sectional study among university students revealed that students who are not stressed consume more energy and fatty foods compared with those who are stressed when comparing the differences in nutritional intake (38).

Perceived stress has been related to unhealthy eating patterns in both men and women, with reports indicating that women have an increased preference for snacks, biscuits, and sweets, while men have an increased preference for fast food and meat (12). Another original study found that women are more likely than men to increase their food intake when stressed, and experimentally demonstrated that stress causes food choices to shift from healthy low-fat foods to less healthy high-fat foods (39). In the present study, we also demonstrated sex differences related to food choices when under stress. Previous studies found that eating healthy foods such as vegetables and fresh fruit is negatively associated with perceived levels of stress in both men and women (40), which is slightly different from our findings. In our study, the association between a preference for fruit and perceived levels of stress was not significant after adjusting confounders in men, indicating the need for further exploration in different populations. Compared to men, women are more health-conscious and consume a larger variety of foods; therefore, they are more prone to lack of restraint when stress eating (41). Accordingly, when stress reduces the inhibitory effect induced by unhealthy high-energy foods, women are inclined to allow themselves to consume them (39). Women are more sensitive to emotion-centered coping methods, distracting themselves from feelings of stress through emotional eating, while men tend to cope with stress in a problem-centered way (22). Interestingly, the current analysis showed that alcohol use was associated with lower perceived levels of stress. A study illustrated that moderate alcohol consumption reduces stress-related neural activity; however, the chronic neurobiological effects of alcohol on stress are uncertain (42).

Overall, our study provides preliminary evidence of the association between perceived levels of stress and self-reported food preference. Further longitudinal studies are required to elucidate the effects of self-reported food preferences on changes in perceived levels of stress. In future research, individuals should be provided more information on the effects of stress on food intake and on foods that may alleviate stress to help them to adopt suitable eating behaviors to combat stress.

### Strengths and Limitations

Our study has several strengths. First, the large sample size increases the generalizability of our findings. Second, all the data used in this study are based on highly reliable CHNS records. Moreover, we controlled for a variety of potential confounders during data analyses. However, the results of our study should be interpreted considering certain limitations. First, causality cannot be inferred owing to the cross-sectional design. Second, the large excluded sample also should be noted. Third, the PSS-14 is a self-report scale and the CHNS only assessed perceived stress levels once, which may have introduced measurement errors and cannot reflect long-term conditions. Moreover, differences may exist between eating preferences and eating behaviors. Finally, the dietary information of the included participants—gathered for the 2015 CHNS—was not publicly available, which limited the exploration of the relationship between perceived level of stress and dietary behavior.

## Conclusion

The present population-based study reported strong associations between perceived level of stress and self-reported food preferences among Chinese adults. Further exploration of these associations using a longitudinal design in different populations is warranted. Dietary behavior should be considered in future studies. Moreover, the current findings provide valuable evidence inform that the future researches should consider the effects of stress on food intake and on foods, which may alleviate stress to help them to adopt suitable eating behaviors to combat stress.

## Data Availability Statement

The datasets presented in this study can be found in online repositories. The names of the repository/repositories and accession number(s) can be found below: https://www.cpc.unc.edu/projects/china.

## Ethics Statement

The studies involving human participants were reviewed and approved by University of North Carolina at Chapel Hill and the Chinese Center for Disease Control and Prevention. The patients/participants provided their written informed consent to participate in this study.

## Author Contributions

BC and XG had full access to all the data in the study and take responsibility for the integrity of the data. BC, FY, and RL: study concept and design. RL and BC: acquisition or interpretation of data and drafting of the manuscript. All authors: critical revision of the manuscript for important intellectual content.

## Funding

This work was sponsored by National Natural Science Foundation of China (No. 32071046) and Chongqing Natural Science Foundation (No. cstc2020jcyj-msxmX1065). The funding agents had no role in the design and conduct of the study; collection, management, analysis, interpretation of the data; preparation, review, or approval of the manuscript.

## Conflict of Interest

The authors declare that the research was conducted in the absence of any commercial or financial relationships that could be construed as a potential conflict of interest.

## Publisher's Note

All claims expressed in this article are solely those of the authors and do not necessarily represent those of their affiliated organizations, or those of the publisher, the editors and the reviewers. Any product that may be evaluated in this article, or claim that may be made by its manufacturer, is not guaranteed or endorsed by the publisher.
