# Supplementary material for: Association Between Perceived Levels of Stress and Self-Reported Food Preferences Among Males and Females: A Stated Preference Approach Based on the China Health and Nutrition Survey
Source: Front Public Health. 2022 Mar 25;10:850411. doi: 10.3389/fpubh.2022.850411 (PMC8989958; doi:10.3389/fpubh.2022.850411)
Supplement: Supplementary file 1 [file Table_1.DOCX]

**Supplemental Table 1. The comparison of low and high perceived stress in different** **basic characteristics of participants**

| **Variables** | | **Perceived stress** | | **χ²** | **p-value** |
| --- | --- | --- | --- | --- | --- |
|  |  | **Low** | **High** |  |  |
| **Age** (years) | |  |  |  |  |
|  | 18-40 | 1008 (23.7) | 988 (24.9) | 5.20 | 0.074 |
|  | 41-59 | 1942 (45.7) | 1848 (46.6) |  |  |
|  | >=60 | 1304 (30.7) | 1126 (28.2) |  |  |
| **Sex** (n, %) | |  |  |  |  |
|  | male | 2155 (50.7) | 2042 (51.4) | 0.64 | 0.425 |
|  | female | 2099 (49.3) | 1920 (48.5) |  |  |
| **Education** (n, %) | |  |  |  |  |
|  | Grad from primary | 765 (18.0) | 836 (21.1) | 38.46 | <0.0001 ^a^ |
|  | Lower middle school degree | 1555 (36.6) | 1569 (39.6) |  |  |
|  | Upper middle school degree | 736 (17.3) | 650 (16.4) |  |  |
|  | Technical or vocational degree | 453 (10.7) | 341 (8.6) |  |  |
|  | University or college degree and above | 745 (17.5) | 566 (14.3) |  |  |
| **Urban** | |  |  |  |  |
|  | city, town or county capital city | 2230 (52.4) | 2260 (57.0) | 17.67 | <0.0001 |
|  | suburban or rural village | 2024 (47.6) | 1702 (43.0) |  |  |
| **Weight category** | |  |  |  |  |
|  | Underweight | 172 (4.1) | 190 (4.8) | 7.55 | 0.056 |
|  | Normal | 1923 (45.2) | 1779 (44.9) |  |  |
|  | Overweight | 1511 (35.5) | 1456 (36.8) |  |  |
|  | Obese | 648 (15.2) | 537 (13.6) |  |  |
| **Smoking status** | |  |  |  |  |
|  | Yes | 967 (23.0) | 883 (22.5) | 0.19 | 0.66 |
|  | No | 3246 (77.0) | 3034 (77.5) |  |  |
| **Alcohol consumption** | |  |  |  |  |
|  | Yes | 1233 (30.2) | 1024 (27.1) | 8.93 | 0.003 |
|  | No | 2851 (69.8) | 2749 (72.9) |  |  |
| **Diabetes** | |  |  |  |  |
|  | Yes | 218 (5.4) | 164 (4.4) | 4.16 | 0.041 |
|  | No | 3859 (94.6) | 3602 (95.6) |  |  |
| **Hypertension** | |  |  |  |  |
|  | Yes | 697 (17.1) | 557 (14.8) | 7.64 | 0.006 |
|  | No | 3384 (82.9) | 3209 (85.2) |  |  |

**Supplemental Table 2. The results of self-reported food preferences survey**

| **Food Preference, n (%)** | **Fast food** | **Salty snack food** | **Fruits** | **Vegetables** | **Soft/sugared drinks** |
| --- | --- | --- | --- | --- | --- |
| Dislike very much | 1656 (20.2) | 1300 (15.8) | 86 (1.1) | 57 (0.7) | 676 (8.2) |
| Dislike somewhat | 4676 (56.9) | 4731 (57.6) | 438 (5.3) | 180 (2.2) | 3698 (45.0) |
| Neutral | 1183 (14.4) | 1416 (17.2) | 1731 (21.1) | 1243 (15.1) | 2352 (28.6) |
| Like somewhat | 394 (4.8) | 529 (6.4) | 5541 (67.4) | 6126 (74.6) | 1237 (15.1) |
| Like very much | 15 (0.2) | 29 (0.4) | 370 (4.5) | 562 (6.8) | 72 (0.9) |
| Does not eat this food | 292 (3.6) | 211 (2.6) | 50 (0.6) | 48 (0.6) | 181 (2.2) |

**Supplemental Table 3.** Logistic regression analysis of food preferences between low and high perceived stress level

| **Food preferences** | **UOR ^a^** | **95% CI** | ***z*** | ***p*-value** | **AOR ^b^** | **95% CI** | ***z*** | ***p*-value** |
| --- | --- | --- | --- | --- | --- | --- | --- | --- |
| **Fast food** | 1.18 | 1.11, 1.25 | 5.45 | <0.0001 | 1.20 | 1.12, 1.28 | 5.47 | <0.0001 |
| **Salty snack food** | 1.19 | 1.12, 1.26 | 5.97 | <0.0001 | 1.20 | 1.13, 1.28 | 5.84 | <0.0001 |
| **Fruits** | 0.74 | 0.69, 0.79 | -9.28 | <0.0001 | 0.74 | 0.69, 0.79 | -8.68 | <0.0001 |
| **Vegetables** | 0.62 | 0.57, 0.67 | -12.12 | <0.0001 | 0.63 | 0.58, 0.68 | -11.2 | <0.0001 |
| **Soft/sugared drinks** | 1.16 | 1.10, 1.22 | 5.75 | <0.0001 | 1.14 | 1.08, 1.20 | 4.85 | <0.0001 |

^a^ Calculated by an unconditional logistic regression model.

^b^ Adjusted OR and 95%CI were calculated by an unconditional logistic regression model adjusting for age, sex, education, urban status, BMI, smoking status, alcohol consumption, diabetes and hypertension.
